# MicroRNA basis of physiological hypertrophy

**DOI:** 10.3389/fgene.2013.00253

**Published:** 2013-11-26

**Authors:** Siyi Fu, Ran Zhuo, Mengchao Yao, Jiawen Zhang, Hanling Zhou, Junjie Xiao

**Affiliations:** ^1^Regeneration Lab, School of Life Science, Shanghai UniversityShanghai, China; ^2^Innovative Drug Research Center of Shanghai UniversityShanghai, China; ^3^Experimental Center of Life Sciences, School of Life Science, Shanghai UniversityShanghai, China

**Keywords:** microRNAs, pathological hypertrophy, physiological hypertrophy, proliferation, heart failure

Cardiac hypertrophy is a distinguished feature of several physiological and pathological remodeling (Frey et al., 2004). Pathological hypertrophy is commonly seen in patients with heart injury or stress, such as myocardial infarction, hypertension, and valve disease (Frey et al., 2004). Physiological hypertrophy is typically induced by exercise or pregnancy. Interestingly, physiological hypertrophy is generally accepted as an adaptive beneficial response while pathological hypertrophy can ultimately decompensate to heart failure, a common, costly, disabling, and deadly disease. Thus, dissecting the mechanisms for physiological hypertrophy will help identify novel effective therapies for a large spectrum of cardiovascular diseases (Da Costa Martins and De Windt, 2012).

Distinct underlying signaling pathways for physiological and pathological hypertrophy have been identified (Maillet et al., 2013). The classic pathway for physiological hypertrophy is IGF-1/PI3K (p110a)/Akt1 while the key one for pathological hypertrophy is AngII (ET-1)/Gaq/Calcineurin/NFAT (Maillet et al., 2013). MicroRNAs (miRNAs, miRs) are a novel class of non-coding RNAs with 20–24 length of base, which posttranscriptional regulates gene expression via base-pairing with complementary sequences within mRNA (Xiao et al., 2012). It is estimated that over 1000 miRNAs were encoded by the human genome. Individual miRNAs can regulate several target genes while one gene can also be regulated by several miRNAs. Being a center player of gene regulation, miRNAs participate in many essential biological processes, including proliferation, differentiation, apoptosis, necrosis, autophagy, and stress responses (Xiao et al., 2012; Kumarswamy and Thum, 2013). Due to these multiple roles, it is naturally that miRNAs are critical in the development of various heart diseases, such as hypertrophy, heart failure, acute myocardial infarction, and arrhythmia (Xiao et al., 2011; Kumarswamy and Thum, 2013). In addition, circulating miRNAs have also been indicated to be promising biomarkers for cardiovascular diseases (Xu et al., 2012). Among them, pathological hypertrophy is the most widely studied one. Accumulating evidence has indicated that a lot of miRNAs such as miR-1, miR-133, miR-26, miR-9, miR-98, miR-29, miR-199a, miR-199b, miR-208, miR-23a, miR-499, miR-21, and mir-19b contribute to pathological hypertrophy (Da Costa Martins and De Windt, 2012). Some distinguished reviews have summarized it in detail (Da Costa Martins and De Windt, 2012; Ellison et al., 2012).

Unlike pathological hypertrophy, only a little studies described how miRNAs response to physiological hypertrophy (Soci et al., 2011; Diniz et al., 2013). It has been reported that miR-1, miR-133, mir-29c, miR-27a, mir-27b, and miR-143 response to physiological hypertrophy (Soci et al., 2011; Da Costa Martins and De Windt, 2012; Ellison et al., 2012). However, these miRNAs are either compensatory or lack of direct evidence for regulating cell size. Thus, these results might only set the beginning of filling the gap in miRNAs and physiological hypertrophy. Although these studies have suggested some potential roles of miRNAs in physiological hypertrophy, more functional studies are highly needed to establish miRNAs as contributors for physiological hypertrophy.

Traditionally, the adult mammalian heart is recognized as a post-mitotic organ with no regenerative capacity for cardiomyogenesis (Rosenzweig, 2012). Recently, resident endogenous cardiac stem-progenitor cells (eCSCs) in the adult heart challenged this dogma (Rosenzweig, 2012). Moreover, adult cardiomyocytes have also been reported to proliferate in response to specific stimuli (Rosenzweig, 2012). Two studies regarding physiological hypertrophy are of great importance (Boström et al., 2010; Waring et al., 2012). A transcriptional factor named CEBPB has been found to be down-regulated with exercise and reduction of CEBPB induces cardiomyocyte hypertrophy and proliferation (Boström et al., 2010). Further studies show that CEBPB promotes cardiomyocyte proliferation via increasing CITED4 (Boström et al., 2010). This study indicates that besides the generally accepted idea that physiological hypertrophy is solely due to the hypertrophy of existing cardiomyocytes, physiological hypertrophy also has the phenotype of new cardiomyocytes formation (Boström et al., 2010). A more recent study shows that c-Kit positive eCSCs increases their number and activated state in exercise-induced physiological hypertrophy, indicating that c-Kit positive eCSCs might be a source of new cardiomyocyte formation (Waring et al., 2012). Therefore, myocyte-restricted lineage tracing studies are highly needed to definitively unravel this question. Anyway, both studies indicate that it is necessary to check miRNAs roles in promoting new cardiomyocyte formation either in cardiomyocytes or in eCSCs in physiological hypertrophy.

With the strategies for checking myocyte hypertrophy and new cardiomyocyte formation, the miRNA basis of physiological hypertrophy will be revealed, which will help develop a miRNA-based therapy for heart failure.
